# Pomalidomide mitigates neuronal loss, neuroinflammation, and behavioral impairments induced by traumatic brain injury in rat

**DOI:** 10.1186/s12974-016-0631-6

**Published:** 2016-06-28

**Authors:** Jin-Ya Wang, Ya-Ni Huang, Chong-Chi Chiu, David Tweedie, Weiming Luo, Chaim G. Pick, Szu-Yi Chou, Yu Luo, Barry J. Hoffer, Nigel H. Greig, Jia-Yi Wang

**Affiliations:** Graduate Institute of Medical Science, College of Medicine, Taipei Medical University, 250 Wu-Hsing St., Taipei, 110 Taiwan; Department of Nursing, Hsin Sheng Junior College of Medical Care and Management, Taoyuan, Taiwan; Department of General Surgery, Chi Mei Medical Center, Tainan and Liouying, Taiwan; Drug Design & Development Section, Translational Gerontology Branch, Intramural Research Program, National Institute on Aging, National Institutes of Health, Baltimore, USA; Department of Anatomy and Anthropology, Sackler School of Medicine and Sagol School of Neuroscience, Tel Aviv University, Tel Aviv, Israel; Graduate Program on Neuroregeneration, College of Medical Science and Technology, Taipei Medical University, Taipei, Taiwan; Department of Neurosurgery, Case Western Reserve University School of Medicine, Cleveland, OH USA; Department of Physiology, College of Medicine, Taipei Medical University, 250 Wu-Hsing St., Taipei, 110 Taiwan

**Keywords:** Pomalidomide, Thalidomide, Traumatic brain injury, Controlled cortical impact, Tumor necrosis factor-α, Interleukin-1β, Interleukin-6, Glutamate excitotoxicity, Neuronal apoptosis, Neuroinflammation

## Abstract

**Background:**

Traumatic brain injury (TBI) is a global health concern that typically causes emotional disturbances and cognitive dysfunction. Secondary pathologies following TBI may be associated with chronic neurodegenerative disorders and an enhanced likelihood of developing dementia-like disease in later life. There are currently no approved drugs for mitigating the acute or chronic effects of TBI.

**Methods:**

The effects of the drug pomalidomide (Pom), an FDA-approved immunomodulatory agent, were evaluated in a rat model of moderate to severe TBI induced by controlled cortical impact. Post-TBI intravenous administration of Pom (0.5 mg/kg at 5 or 7 h and 0.1 mg/kg at 5 h) was evaluated on functional and histological measures that included motor function, fine more coordination, somatosensory function, lesion volume, cortical neurodegeneration, neuronal apoptosis, and the induction of pro-inflammatory cytokines (TNF-α, IL-1β, IL-6).

**Results:**

Pom 0.5 mg/kg administration at 5 h, but not at 7 h post-TBI, significantly mitigated the TBI-induced injury volume and functional impairments, neurodegeneration, neuronal apoptosis, and cytokine mRNA and protein induction. To evaluate underlying mechanisms, the actions of Pom on neuronal survival, microglial activation, and the induction of TNF-α were assessed in mixed cortical cultures following a glutamate challenge. Pom dose-dependently ameliorated glutamate-mediated cytotoxic effects on cell viability and reduced microglial cell activation, significantly attenuating the induction of TNF-α.

**Conclusions:**

Post-injury treatment with a single Pom dose within 5 h significantly reduced functional impairments in a well-characterized animal model of TBI. Pom decreased the injury lesion volume, augmented neuronal survival, and provided anti-inflammatory properties. These findings strongly support the further evaluation and optimization of Pom for potential use in clinical TBI.

## Background

Traumatic brain injury (TBI) is the leading cause of death and long-term disability in the developed world. Annually, an estimated ten million people suffer a TBI event worldwide [1, 2]. Projections indicate that TBI will comprise the third largest portion of the total global disease burden by 2020 [1]. Within the USA, an estimated 1.7 million people per year sustain a TBI, and approximately 5.3 million people live with a TBI-induced disability [3, 4]. Within Taiwan, an estimated 52,000 TBIs occur annually, and up to 25 % of them are fatal [5].

With increases in survival following initial injury, TBI can result in substantial and lifelong cognitive, physical, and behavioral impairments that require long-term access to health care and disability services [6, 7]. Specifically, some 70–90 % of patients continue to manifest prolonged and often permanent neurocognitive dysfunctions that can substantially impact their performance and/or quality of life. Additionally, epidemiological evidence points to an elevated prevalence of military veteran survivors of TBI developing dementia in later life [8], with an increased risk of Alzheimer’s disease, Parkinson’s disease, and amyotrophic lateral sclerosis [9]. In light of the lack of any available therapeutic [10], it is important to understand the mechanisms underpinning TBI in order to develop effective strategies that can attenuate the neuronal dysfunction and loss that ensues from head injury.

TBI-associated brain damage can be segregated into two key phases. First, an initial primary damage phase occurs at the moment of insult and includes contusion and laceration, diffuse axonal injury, and intracranial hemorrhage that results in immediate (necrotic) cell death [11, 12]. This is followed by an extended second phase that encompasses cascades of biological processes initiated at the time of injury that may persist over subsequent days, weeks, and, possibly, months, consequent to ischemia, neuroinflammation, glutamate toxicity, altered blood-brain barrier permeability, oxidative stress, astrocyte reactivity, cellular dysfunction, and apoptosis [13–16]. As secondary brain injury may be reversible, to develop an effective treatment, it is imperative to understand the biological cascades that drive the delayed secondary phase subsequent to TBI [10, 11].

TNF-α, a key pro-inflammatory cytokine, is synthesized and released by activated microglial cells. Once released and if not appropriately time-dependently restored to a non-activated state, microglial dysregulated TNF-α generation can initiate a self-propagating cycle of unchecked inflammation that can drive neuronal dysfunction and disease processes [17]. Pharmacological interruption of this cycle may be of significant benefit for disorders with a neuroinflammatory component. The drug “thalidomide” can lower TNF-α protein levels post-transcriptionally by accelerating degradation of TNF-α messenger RNA (mRNA) [18]. However, it is not a particularly potent TNF-α synthesis inhibitor in vivo and is associated with sedation, serious teratogenic adverse effects, and neurotoxicity at high clinical doses [19]. In the light of our recent studies demonstrating that the novel and more potent TNF-α synthesis inhibitor 3,6′-dithiothalidomide could effectively mitigate the neuronal apoptosis, gliosis, and behavioral impairments instigated by weight drop-induced mild TBI in mice [20] and other neurodegenerative conditions [21, 22], in the present study, we evaluated a clinically available, more potent amino thalidomide analog, pomalidomide (Pom), in a moderate to severe TBI model. Pom is an immunomodulatory agent with a reported TNF-α inhibitory action of up to 50,000-fold greater than thalidomide [23], whose additional suppressive effects on angiogenesis and tumor cell proliferation underpin its use and efficacy in the treatment of multiple myeloma and other cancers [24].

In the present study, we assessed the effects of the US Food and Drug Administration (FDA)-approved drug Pom on mediators of secondary injury in a rodent model of a penetrating brain injury, resulting from controlled cortical impact. A single Pom treatment provided favorable outcomes across a broad series of acute histological and functional measures at a clinically relevant dose [25]. This study strongly supports further investigation of Pom in additional models of TBI to evaluate potential translational applications in human TBI.

## Methods

### In vivo studies

#### Animal model of TBI and drug administration

All animals were treated in accordance with the International Guidelines for animal research. The study design was approved by the Animal Ethics Committee of Taipei Medical University. All procedures undertaken were covered under the following animal study protocol: LAC-2015-0051. Animals were housed in a temperature (21–25 °C)- and humidity (45–50 %)-controlled room with a 12-h light/dark cycle with *ad libitum* access to pellet chow and water. Male Sprague–Dawley rats (250–300 g, body weight) were anesthetized with 8 % chloral hydrate (400 mg/kg; Sigma, St. Louis, MO) and placed in a stereotaxic frame. A 5-mm craniotomy was performed over the left parietal cortex, centered on the coronal suture, and 3.5 mm lateral to the sagittal suture. TBI was induced using a controlled cortical impact instrument with a rounded metal tip (5-mm diameter). The tip was propelled at a velocity of 4 m/s, and it created a deformation 2 mm below the dura, as described previously [26–28]. Sham animals received anesthesia and craniotomy but no TBI. To control for fluctuations in rodent body temperature due to anesthesia or TBI or to drug interactions, animal body temperature was monitored by the use of a rectal probe and was maintained at 37.0 ± 0.5 °C using a heated pad. Thereafter, animals were placed in a heated cage to maintain body temperature while recovering from anesthesia. To further assess for potential changes in body temperature induced by Pom and anesthesia, a series of sham and TBI animals were administered with both doses of drug and their body temperature was likewise monitored over a 3-h interval.

**Fig. 1:**
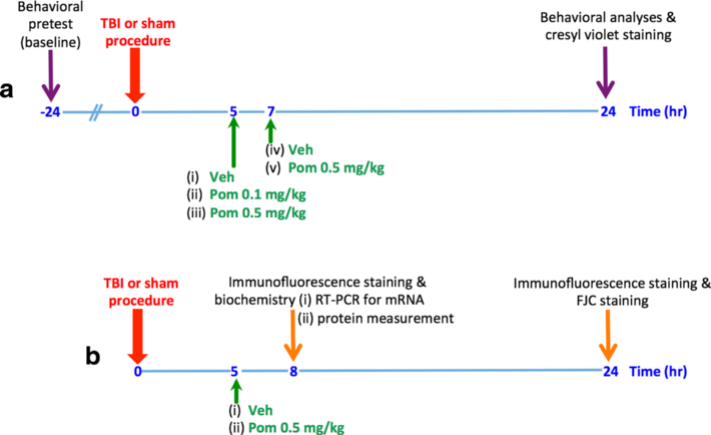
Scheme of animal study design. **a** For contusion volume (or lesion) and behavioral analyses, animals were submitted to TBI or a sham procedure at time zero; thereafter, they were dosed with either vehicle (veh) or pomalidomide (Pom) at either 5 or 7 h later. Behavioral analyses were performed after 24 h, and thereafter, animals were euthanized for evaluation of contusion volume. **b** For morphological evaluation of neurodegeneration and apoptosis or biochemical measurement of cytokine mRNA and protein levels, animals were subjected to TBI or a sham procedure at time zero; thereafter, they were dosed with either vehicle (veh) or pomalidomide (Pom) at 5 h and were euthanized at either 8 or 24 h. For each group, *n* = 5

All animals were randomly assigned into three main groups: sham injury (i.e., control animals with craniotomy but without the TBI procedure (*n* = 5)), TBI plus drug vehicle (veh) (i.e., animals challenged with TBI and treated with vehicle not containing Pom (*n* = 5)), and Pom-treated TBI animals (i.e., animals subjected to TBI and then treated with Pom). As illustrated in Fig. 1a, these TBI + Pom treatment animals were then subdivided into several groups based on dose and treatment time: Pom 0.5 mg/kg administered intravenously at 5 or 7 h (*n* = 5 for both groups) or Pom 0.1 mg/kg administered intravenously at 5 h (*n* = 5). These animal group numbers were selected based upon our prior studies where an *n* = 5 was sufficient to show statistically significant differences in relation to different treatment effects [28]. Figure 1b depicts the time points for morphological evaluation of contusion (or lesion) volume, neurodegeneration, and apoptosis or biochemical measurement of cytokine mRNA and protein levels. Our selected treatment times of 5 and 7 h were based on our prior studies indicating that the secondary phase of neuronal apoptosis following CCI TBI can be inhibited if appropriate treatment is initiated within this time window [28]. Finally, our selected doses of Pom were based on dose translation from rat to human studies, based on normalization of body surface area between species in accord with FDA guidelines.

### Pom synthesis and vehicle

Pom, 4-amino-2-(2,6-dioxopiperidin-3-yl)isoindoline-1,3-dione, was synthesized from 3-nitrophthalic anhydride and 2,6-dioxopiperidine-3-ylamine trifluoroacetate by reflux in acetic acid under a nitrogen atmosphere to afford 2-(2, 6-dioxopiperidine-3-yl)-4-nitrophthalimide. This product was characterized, purified, and then reacted with palladium on activated carbon in methanol under an atmosphere of hydrogen to generate Pom. Filtration and purification by chromatography followed by chemical characterization verified the structure of the final product as Pom with a purity of >99.5 %. The drug vehicle comprised of a mixture of dimethyl sulfoxide (DMSO, Sigma, St. Louis, MO) in normal saline (10 % DMSO in saline) that was administered by i.v. injection.

### TBI and TBI plus Pom effects on injury volume

To measure the volume of TBI-induced injury in the left parietal cortex 24 h after TBI, cresyl violet-stained sections were digitized and analyzed using a ×1 objective and evaluated using ImageJ (National Institutes of Health, Bethesda, MD). The injury volume measurement was performed as previously described [26]. The injury area was calculated from all images of cresyl violet-stained sections from the brain region of interest. The volume was computed by adding the areas and was multiplied by the inter-slice distance (in this situation 500 μm). Hemisphere tissue loss was expressed as a percentage that was calculated by the use of the following formulae: [(contralateral hemispheric volume − ipsilateral hemispheric volume) / (contralateral hemispheric volume) × 100 %], as previously reported [29]. The percentage of tissue loss was determined for each animal, and a mean value was then generated and used to create a plot of injury volume per treatment group; the percentage of tissue loss was used for statistical analysis.

### TBI and TBI plus Pom effects on numbers of degenerating cortical neurons

Schmued and Hopkins initially described an immunohistochemical method that was able to identify degenerating neurons by the use of an anionic fluorescein derivative stain called Fluoro Jade B [30]. In the present study, we used a similar yet more sensitive agent, Fluoro Jade C (FJC) to identify TBI-induced degenerating neurons [31]. Specifically, we used a Fluoro-Jade C from a ready-to-dilute staining kit (Biosensis, TR-100-FJ), with some modification [28]. Brain sections from the different treatment groups were de-paraffinized, followed by a rehydration step in distilled water for 2 min. The slides were incubated in a 1 in 15 diluted solution of potassium permanganate for 10 min, rinsed in distilled water for 2 min and incubated in a 1:25 diluted FJC solution for 30 min. The slides were then washed and mounted on coverslips with Vecta-shield mounting medium (Vector Laboratories, Burlingame, CA, USA). All sections were observed and photographed with a fluorescence microscope; slides were exposed to an excitation light source of 450–490 nm with the resulting emission at 521 nm recorded and analyzed. The numbers of FJC-positive cells were counted in five randomly selected fields per slide by means of SPOT image analysis software (Diagnostic Instruments, Sterling Heights, MI). The numbers of FJC-positive cells observed on the slides from the different treatment groups were counted and used to generate a mean number per treatment group. The mean numbers of FJC-positive cells were then plotted and used for statistical analysis.

### Assessment of motor function and neurological outcome following TBI and Pom-treated TBI

All analyses were performed in a non-biased, blinded manner. Where a baseline measurement or specific pre-injury training was required for motor function analysis, observations/trainings were undertaken prior to and 24 h after TBI. Assessments included an (i) elevated body swing test, (ii) a beam walk test, (iii) a tactile adhesive removal test, and (iv) a neurological severity score (NSS). As TBI was performed on the left side of the brain, any resulting motor functional impairments developed on the right side.

### Asymmetrical motor function

Body asymmetry was quantitatively analyzed by the use of the elevated body swing test (EBST), as initially described by Borlongan and co-workers [32]. Briefly, animals were examined for lateral movement/turning when their bodies were suspended 10 cm above the testing table. The animals were lifted from the table while held by the base of the tail. A left/right swing was counted when the head/torso of the animal moved more than a 10° angle from its vertical axis after elevation. The frequency of the left/right swings was scored across 20 consecutive trials and expressed as a percentage calculated as follows: [(number of right-biased swings/ the total number of swings) × 100 %]. An uninjured animal shows an equal frequency to swing to either the left or right side. The number of contralateral rotations was determined and used to generate a mean number of rotations for each treatment group, which then was statistically analyzed.

### Fine motor coordination

TBI-induced disturbance in fine motor coordination was assessed by the use of the beam walk test [33]. Each animal was placed onto a brightly illuminated platform. Due to a rodent’s inherent preference for a darkened enclosed environment, as compared to an open illuminated environment, each animal was allowed to move freely towards and to enter a darkened goal box [27]. The beam was constructed with the following dimensions: 2.5 cm × 122.0 cm. The time taken for each animal to traverse the beam to reach the goal box was recorded (with the caveat that total time was not to exceed 60 s). Five trials were recorded for each animal before TBI and then at 24 h after TBI. The mean times to traverse the beam were calculated, and a plot was generated to evaluate treatment effects on beam walk times; these times were used for statistical analysis.

### Somatosensory function assessment

A tactile removal test was used to evaluate somatosensory function; this test measures the ability of the animal to perform sensitive paw to mouth movements and mouth, paw dexterity. Essentially, two small adhesive stickers were used as bilateral tactile stimuli that were placed on the distal–radial region on the wrist of each forelimb [27, 34]. Animals were pre-trained daily for 3 days before TBI, and the time required (no longer than 3 min) for the animal to remove the sticker from the forelimb was recorded for five trials undertaken 24 h after TBI. The mean time taken to remove the stickers from the last trial was used to generate a plot displaying the latency time of the sticker removal from each paw; the times were used for statistical analysis.

### Neurological severity score

To compare any neurological deficit severities in TBI animals, a modified neurological severity score (mNSS) was performed. The mNSS included a composite of motor, sensory, reflex, and balance tests [27]. One point was scored for the inability to perform the test or for the lack of a tested reflex. Accordingly, the higher the mNSS score the more severe the injury; neurological function was graded on a scale of 0–18 (a normal animal score being 0 and a maximally impaired animal score being 18). The overall score generated for each animal was determined, and then, a mean for each treatment group was calculated and used to create a plot displaying the average mNSS for each treatment group. The scores were used then for statistical analysis.

### TBI and TBI plus Pom effects on numbers of apoptotic cortical neurons

Immunohistochemical analyses of the level of TBI-induced apoptosis were performed as has been previously described [28]. To assess for early detection of apoptosis, a series of animals were euthanized at 8 h (*n* = 5) in addition to 24 h (*n* = 5) post-TBI (Fig. 1b). Cellular changes observed to occur during apoptosis include the externalization of the phospholipid phosphatidylserine (PS). Specifically, the translocation of PS, from the inner membrane to the outer membrane, is used routinely as a marker in the early stages of apoptosis. Annexin V binds strongly with PS, and its detection has been widely used for imaging of apoptosis both in vitro and in vivo; thus, to label external PS, we stained for the protein annexin V [35, 36]. In brief, all sections were dried and then rehydrated in phosphate-buffered saline (PBS). The sections were blocked for 1 h in a 5 % bovine serum albumin (BSA, Sigma, St. Louis, MO) PBS-based buffer after which they were incubated with the appropriate primary antibodies. To detect mature neurons, a monoclonal anti-NeuN antibody (Millipore; 1:500) was used. To detect annexin V, an anti-annexin V antibody was used (Abcam; 1:500). All primary antibody incubations took place at 4 °C, overnight. Primary antibody detection was undertaken with fluorescent secondary antibodies: Alexa Fluor® 488 goat anti-rabbit IgG (1:200, Jackson ImmunoResearch, West Grove, PA) and Alexa Fluor® 594 anti-mouse IgG (1:200 dilution, Jackson ImmunoResearch, West Grove, PA). Tissue samples were incubated with secondary antibodies at room temperature for 2 h and were then treated with Mounting Medium H-1000 (Vector Laboratories, Burlingame, CA, USA). The numbers of dual stained NeuN- and annexin V-positive cells were then counted in five randomly selected fields per slide in five slides, by means of SPOT image analysis software (Diagnostic Instruments, Sterling Heights, MI). Numbers of NeuN- and annexin V-positive neurons observed on the slides from the different treatment groups were used to calculate a mean number of apoptotic neurons per animal. This value was then used to generate a mean number per treatment group. The mean numbers of double-positive neurons were plotted and statistically analyzed.

### TBI and TBI plus Pom effects on mRNA levels of pro-inflammatory cytokines

Total RNA was prepared from cortical tissue (approximately 50 mg) 8 h post-injury (*n* = 5) by the use of TRIzol reagent (Invitrogen, Life technologies, Carlsbad, CA, USA); the protocol used was as recommended by the manufacturer. The quality and concentration of the total RNA were determined by measuring absorbance at 260 and 280 nm wavelengths. Total RNA (3 μg) underwent reverse transcription (RT) that was followed by a complementary DNA (cDNA) synthesis step. The RT step was performed using a total reaction volume of 20 μl with ReverTra Ace® set number PU-TRT-100 (Purigo, Taipei, Taiwan). The RT products were used as templates in quantitative (real-time) PCR or were stored at −20 °C. The quantification of target mRNA levels was performed using the qPCR QuantiFast SYBR Green PCR kit (Qiagen) with primers in a Rotor-Gene Q 2plex HRM Platform (Qiagen). Reaction conditions were carried out for 35–40 cycles (5 min at 95 °C, 10 s at 95 °C, and 30 s at 60 °C). The primers were designed using reported cDNA sequences: TNF-α, CTC TTC TCA TTC CCG CTC GTG (forward) and GGA ACT TCT CCT CCT TGT TGG G (reverse); IL-1β, GTT TGA GTC TGC ACA GTT CCC (forward) and CAA CTA TGT CCC GAC CAT TGC (reverse); IL-6, TTC TTG GGA CTG ATG TTG TTG AC (forward) and AAT TAA GCC TCC GAC TTG TGA AG (reverse); and β-actin, GAC CCA GAT CAT GTT TGA GAC CTT C (forward) and GAG TCC ATC ACA ATG CCW GTG G (reverse). Relative transcript expressions of inflammatory target mRNAs were normalized to β-actin, which was used as an internal control. Transcript levels were expressed as values relative to the control group using the comparative cycle threshold (Ct) method.

### Effects of Pom on the TBI or excitotoxicity activation of pro-inflammatory cytokine proteins

Rat cortical tissues were harvested 8 h post-injury (*n* = 5), homogenized in lysis buffer (RIPA, Sigma, St Louis, MO), and centrifuged at 8000*g* for 10 min at 4 °C. The resulting supernatant was collected and stored at −80 °C until the time of analysis of protein levels by specific ELISAs for rat TNF-α, IL-1β, and IL-6 (R&D System (RTA00; RLB00; R6000B)). The sample protein content was determined by the use of the BCA assay (Pierce™ BCA Protein Assay Kit, #23225), and levels of cortical protein were determined by following the manufacturers protocol. Cortical cytokine protein levels were normalized to a pg/mg of tissue unit.

### In vitro studies

#### Pom effects on excitotoxic and oxidative stressors of rat primary cortical cultures

Primary cortical neuronal/glial co-cultures were isolated from the cerebral cortex of 1-day-old neonatal Sprague–Dawley rats, as previously described [37]. These procedures were performed in accordance with the National Institutes of Health Guidelines for the Care and Use of Laboratory Animals and were approved by the Institutional Animal Care and Use Committee of Taipei Medical University, Taipei, Taiwan (animal study protocol number LAC-2015-0051). Rat pup brains were removed immediately following euthanasia and the cerebral cortex placed in ice-cold Hank’s solution (without Ca^2+^ or Mg^2+^). Cortical cells were dissociated and suspended in Dulbecco’s modified Eagle’s medium (DMEM, Gibco BRL, Grand Island, NY, USA) supplemented with 10 % fetal bovine serum (FBS). Thereafter, cells were plated at a density of 5 × 10^5^ cells/ml in 24-well culture plates and then incubated at 37 °C in a humidified incubator (5 % CO_2_, 95 % air). All experiments were performed 13–14 days after the plating (100 % confluency), with *N* = 3 for each treatment and control group.

The percentage of neurons, astrocytes, and microglial cells were assessed by immunostaining. Mature neurons were stained with NeuN (Millipore; 1:500), astrocytes with anti-GFAP (Millipore ab5804; 1:1000), and activated microglia with anti-CD68 antibody (clone ED1, ab31630, abcam 1:500). Under our culture conditions, the neuronal/glial co-cultures comprised of approximately 35 % of neurons, 54 % astrocytes, and fewer numbers of microglia (6 to 8 %), in line with our previous studies [28, 37]. To mimic TBI-induced excitotoxicity stress in vitro, neuronal/glial co-cultures were challenged with glutamate (Sigma, St Louis, MO). Different concentrations of stressor were used to assess the levels of excitotoxicity (not shown). From these pilot studies, 24 h challenge with 100 mM glutamate was observed to be sufficient to cause a non-maximal level of neuronal cellular loss, and hence, this time and concentration were selected for all following experiments. To evaluate potential neuroprotection afforded by Pom against the stressor toxicity, different concentrations of Pom (3 to 100 μM) or vehicle (0.1 % DMSO in PBS) were added 30 min after initiation of a 24-h challenge with glutamate (100 mM).

The effects of Pom treatment on cortical culture media levels of glutamate-induced microglial TNF-α protein generation were determined over time. Pom (100 μM) was administered 30 min after challenge with the stressor, and culture media was harvested for analysis at 3, 6, and 24 h after the stressor challenge. Protein levels of rat TNF-α were measured in culture media by ELISA (R&D System RTA00) according to the manufacturer’s protocol, and the results are expressed as pg/ml.

### Statistical analysis

Comparisons between treatment groups were conducted using one-way ANOVA with a post hoc test. The Bonferroni correction was used for repeated measures. All statistical analyses and bar graph displays were carried out using Sigma Plot and Stat version 2.0 from Jandel Scientific, San Diego, CA. Data are presented as mean ± standard error of the mean (S.E.M.) values, and statistical significance (*p < 0.05, **p < 0.01 or ***p < 0.001) is noted in the legend of each figure.

## Results

### TBI injury in rodent

#### Pom treatment dose- and time-dependently reduced TBI injury volume

**Fig. 2:**
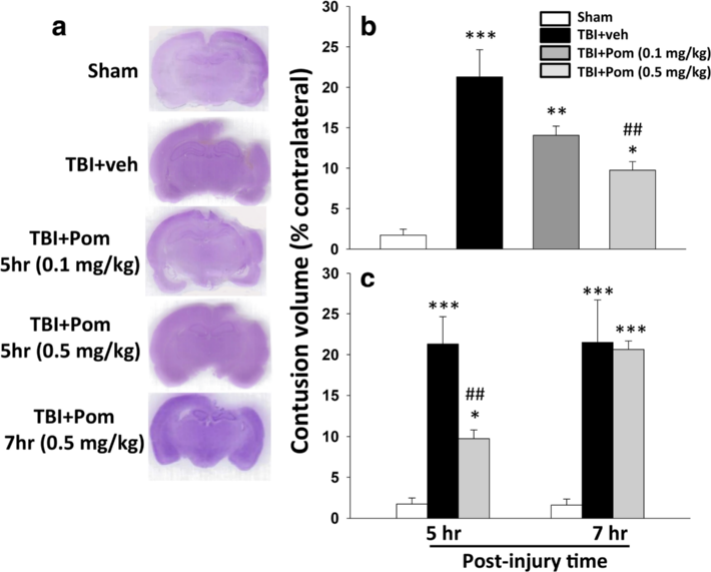
Post-injury administration of pomalidomide (Pom: 0.5 mg/kg, i.v.) at 5 h after TBI significantly reduced contusion volume evaluated at 24 h. A lower Pom dose (0.1 mg/kg i.v.) and longer time window (7 h) reduced efficacy. **a** Representative cresyl violet stained coronal brain sections of the CCI TBI-induced cavity in Sham (control without TBI), TBI-vehicle (TBI + veh), and pomalidomide-treated TBI rats (TBI + Pom) at 24 h post-TBI. **b** The TBI-induced contusion volume evaluated at 24 h was significantly reduced by Pom (0.5 mg/kg), but not by Pom (0.1 mg/kg) treatment. **c** Post-injury administration of Pom (0.5 mg/kg) at 5 h, but not at 7 h, significantly reduced the contusion volume relative to TBI + vehicle (TBI + veh) group. Data are expressed as mean ± S.E.M. (*n* = 5 in each group). *p < 0.05, **p < 0.01, ***p < 0.001 compared with the sham group. ^##^ *p* < 0.01 compared with the TBI + veh group

TBI caused cortical tissue injury that resulted in a loss of volume in the ipsilateral hemisphere (Fig. 2a). The injury volume observed in the TBI + veh animals was in the order of 21 ± 3 % of the contralateral hemisphere volume. Post-injury administration of Pom (0.5 mg/kg, i.v.) at 5 h significantly reduced the injury volume to 10 ± 1 % of the contralateral hemisphere volume. This represented a reduction of the lesion volume in the order of 54 ± 2 % compared to the TBI + veh group (*p* < 0.01). Pom (0.1 mg/kg, i.v.) at 5 h reduced the injury volume to ~14 ± 1 %, representing a 34 ± 2 % reduction that was not significantly different from the TBI + veh group (Fig. 2b).

To evaluate the importance of time dependence of drug administration after TBI to define a window of opportunity, a comparison of the most effective dose of Pom (0.5 mg/kg, i.v.) was made when administered at 5 h compared to 7 h post-TBI. As previously noted, administration of Pom at 5 h significantly reduced the contusion volume. However, delaying administration to 7 h post-TBI resulted in no significant reduction in injury volume with a difference of 1.0 ± 1.0 % compared to the TBI + veh group (Fig. 2c). As Pom proved to be efficacious at reducing the injury volume at 5 h but not at 7 h post-TBI, we chose to study Pom (0.5 mg/kg, i.v.) at 5 h post-TBI for all subsequent experiments.

### Pom treatment reduced TBI-induced neurodegeneration

**Fig. 3:**
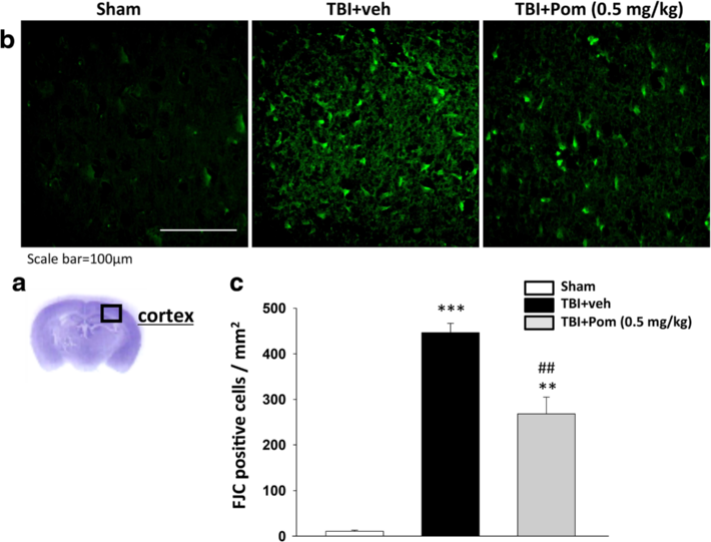
TBI induces neuron degeneration within the contusion region, and treatment with Pom significantly reduced the number of TBI-induced degenerating neurons. **a** Representative cresyl violet stained coronal brain section of the Sham (control without TBI) that shows the area of evaluation. **b** Representative photomicrographs showing the presence of FJC-staining at 24 h in the sham (control without TBI), TBI + veh group, and TBI + Pom administered 5 h post-injury group. **c** Quantitative comparison of mean densities of FJC-positive cells in the cortical contusion area at 24 h post-injury. There was a significant decrease in the number of FJC-positive cells in the TBI + Pom group. Mean ± S.E.M. (*n* = 5 in each group). **p < 0.01, ***p < 0.001 compared with the sham group. ^##^ *p* < 0.01 compared with the TBI + veh group. *Scale bar* = 100 μm

As illustrated in Fig. 3a, numerous FJC-positive cells were observed within the cortical contusion margin at 24 h post-injury. A quantitative summary of FJC-positive cells observed in the different treatment groups is provided in Fig. 3b. There were significant levels of degenerating neurons in the TBI + veh group, as compared to sham animals (468 ± 26/mm^2^, four slides per animal from *n* = 5 animals). In comparison, the Pom-treated TBI group (Pom 0.5 mg/kg, 5 h) had a significantly lower number of degenerating neurons (268 ± 37/mm^2^, four slides per animal from *n* = 5 animals) compared to the TBI + veh group. This difference represents a significant reduction of ~43 % compared to the vehicle treatment group (*p* < 0.01).

### Pom improved multiple functional outcomes as revealed by behavioral evaluation at 24 h after TBI

**Fig. 4:**
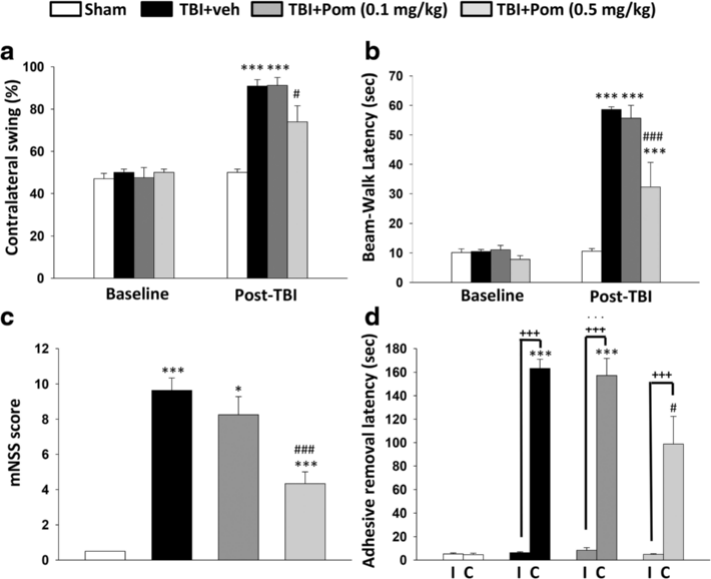
Pom administered at 5 h post-injury improved functional outcomes as revealed by behavioral evaluation at 24 h after TBI. **a** Motor asymmetry measured by elevated body swing test (EBST). TBI-induced deficits were attenuated by Pom (0.5 mg/kg). **b** Motor coordination and balance measured by beam walking test. TBI impairments were attenuated by Pom (0.5 mg/kg). **c** Animals treated with Pom (0.5 mg/kg), overall presented with better neurological severity scores (mNSS), as indicated in the animal groups scores shown in (**c**). **d **TBI induced abnormalities in sensory/motor function that were revealed by a longer latency for the animals to remove an adhesive sticker from their contralateral (**c**) but not ipsilateral (I) forepaw. Treatment with Pom (0.5 mg/kg) significantly attenuated this behavioral deficit. Data represent the mean ± S.E.M. (*n* = 5 in each group). *p < 0.05, ***p < 0.001 versus sham group; ^#^ *p* < 0.05, ^###^ *p* < 0.001 versus TBI + Veh group; ^+++^ *p* < 0.001 versus non-impaired left

As evident in Fig. 4, TBI induced numerous changes in rodent motor function that were attenuated by treatment with Pom. Notably, no changes in body temperature regulation were observed in any sham or TBI animals, whether administered Pom (0.1 or 0.5 mg/kg) or veh at 5 or 7 h following TBI.

Asymmetrical motor function, as evaluated by elevated body swing that combines assessments of tactile/sensory and motor functions [32], was identical prior to TBI across all animals, yet after TBI, significant differences were apparent by 24 h after injury. Specifically, asymmetry was elevated in TBI + veh animals, as compared to sham levels (Fig. 4a), with an increase in the contralateral swing ratio in TBI + veh animals. Pom (0.5 mg/kg) treatment at 5 h post-TBI significantly improved functional deficits by reducing the contralateral swing ratio from 91 ± 3 to 74 ± 8 % (*p* < 0.05), a decline of 17 %.

Fine motor coordination, as evaluated by the beam walk test as a task that combines assessments of motoricity/coordination and anxiety, similarly was identical across groups prior to TBI, yet after TBI, significant impairments were observed at 24 h post-injury (Fig. 4b). Beam walk latency time was elevated following TBI and was significantly different between sham-, TBI + veh-, and Pom (0.5 mg/kg)-treated TBI animals. The Pom group treated at 5 h post-injury demonstrated significantly better performance in beam walking with a latency of 32 ± 8 s (*p* < 0.001) compared to 59 ± 2 s for the TBI + veh animal group.

Modified neurological severity score assessment provided a composite evaluation of a broad series of sensory and motor functions [38] and demonstrated that TBI in the left cortical hemisphere resulted in physiologically relevant functional deficits (Fig. 4c). As compared with the sham, the TBI + veh group had a significantly increased mNSS score (9.6 ± 0.7 units). Post-injury administration of Pom 0.5 mg/kg, but not 0.1 mg/kg, at 5 h mitigated neurological deficits, as revealed by a significantly lower mNSS score (p < 0.001) that was 55 ± 4 % of the TBI + veh group.

 Somatosensory function, as evaluated by the time taken for adhesive sticker-removal from the contralateral paw of rodents subjected to TBI as a task that combines sensory and motor skills, was impaired following TBI (Fig. 4d). Sham animals removed the sticker from both contralateral and ipisilateral paws with equal dexterity and speed. However, the TBI + veh and TBI + Pom groups showed functional deficits, requiring a significantly greater time for removal of stickers from their contralateral paw, compared to the sham group. For the TBI + veh group, the removal time was 163 ± 8 s, as compared to 5 ± 1 s (sham) (*p* < 0.001). TBI + Pom 0.1 and 0.5 mg/kg animal groups took longer (157 ± 14 and 99 ± 24 s, respectively) to remove the contralateral sticker compared to the sham group (5 ± 1 s, *p* < 0.001). However, the TBI + Pom (0.5 mg/kg) animals were less impaired than the TBI + veh group, requiring a significantly shorter time to remove the contralateral sticker (99 ± 24 s compared to 163 ± 8 s; *p* < 0.05 versus TBI + veh group).

### Pom reduced the numbers of apoptotic neurons induced by TBI

**Fig. 5:**
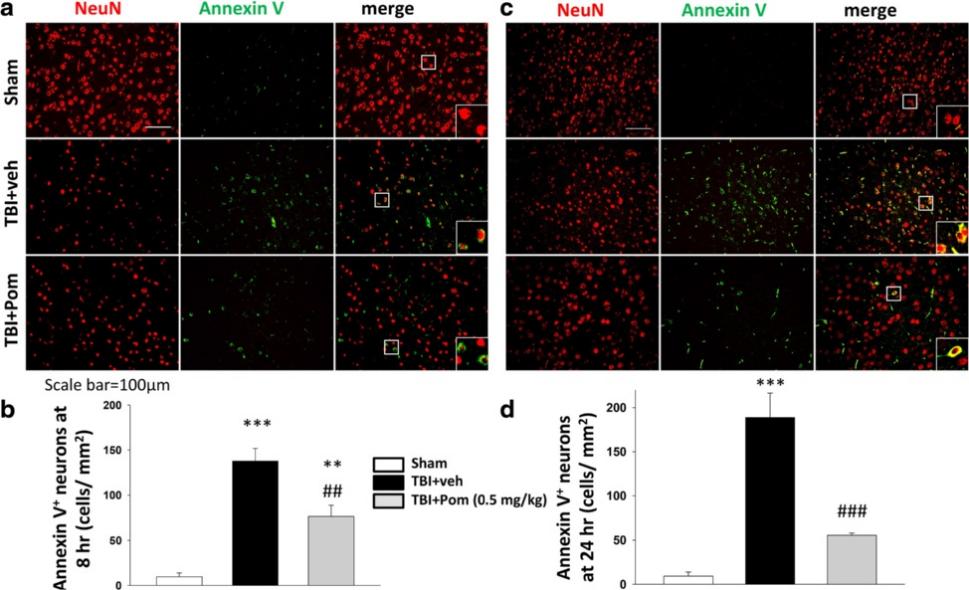
Post-injury administration of Pom at 5 h after TBI decreased annexin V-positive neurons at 8 and 24 h. **a** Immunofluorescence of annexin V and NeuN in cortical brain tissue at 8 h post-injury. Annexin V immunoreactivity (a marker of early apoptosis) is shown in *green*; NeuN (a marker for mature neurons) is shown in *red. Merged red* and *green cells* are shown as *yellow*, which highlights dual labeled cells suggesting an apoptotic neuronal state. Cells illustrated in the *inset box* in the *lower right section of the merged images* are magnified examples of the cells indicated in the smaller observation box. **b** Treatment with Pom significantly reduced the number of dual stained annexin V-positive neurons, indicating a lower incidence of apoptotic neurons. Similar observations are shown for tissues obtained from animals euthanized at 24 h post-TBI (**c**, **d**). TBI induced a significant number of apoptotic neurons at 24 h post-injury, and treatment with Pom significantly reduced the numbers of apoptotic neurons compared with TBI + veh. Mean ± S.E.M. (*n*=5 in each group). **p < 0.01, ***p < 0.001 compared with the sham group. ^##^ *p* < 0.01, ^###^ *p* < 0.001 compared with the TBI + veh group. *Scale bar* = 100 μm

Significant levels of apoptosis occur in the injured cortex after TBI. Initially, we assessed for levels of apoptotic cells at 8 h after TBI. Shown in Fig. 5a are representative cortical sections illustrating numbers of neuronal cells (NeuN, labeled red) and apoptotic-labeled cells (annexin V labeled green). In TBI + veh animals, the numbers of dual stained apoptotic cells (shown in the “merged” image Fig. 5a) were significantly increased at 8 h after injury, as compared with sham animal tissues. Specifically, the number of apoptotic neurons was 137 ± 14 cells/mm^2^, (*p* < 0.001 versus sham) (Fig. 5b). Pom administration reduced the number of dual stained apoptotic neurons to 76 ± 12 cells/mm^2^, thus decreasing the level of neuronal apoptosis by 45 %, as compared with the TBI + veh group (*p* < 0.01; Fig. 5b).

Upon examination of the numbers of TBI-induced apoptotic neurons determined at 24 h after injury (Fig. 5c), we similarly observed a significant number of apoptotic neurons in TBI + veh brain (189 ± 28 cells/mm^2^, *p* < 0.001 versus sham Fig 5d). Treatment with Pom markedly reduced dual stained apoptotic neuron number to 55 ± 2 cells/mm^2^; thereby decreasing the level of neuronal apoptosis by 71 %, as compared with the TBI + veh group (*p* < 0.001; Fig. 5 c, d).

### Pom reduces TBI-induced cortical cytokine mRNA expression and protein abundance

**Fig. 6:**
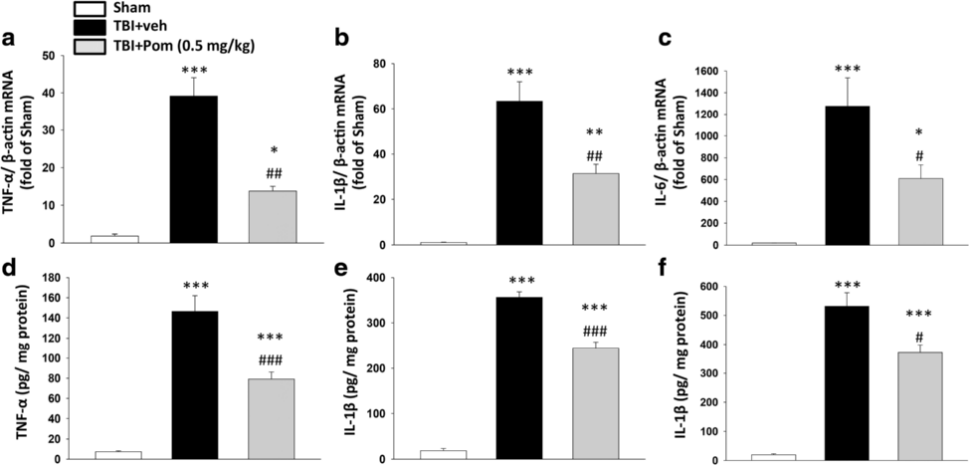
Treatment with Pom 5 h after TBI significantly reduced injury-induced cortical cytokine mRNA expression and protein abundance at 8 h after TBI. TBI-induced marked elevations in mRNA levels of **a** TNF-α, **b** IL-1β, and **c** IL-6. Treatment with Pom (0.5 mg/kg) significantly attenuated the elevation in mRNA transcript levels when compared with TBI + veh levels. Changes in cytokine protein abundance mirrored those of mRNA transcripts (**d**, **e**, **f**). Mean ± S.E.M. (*n* = 5 in each group). *p < 0.05, **p < 0.01, ***p < 0.001 compared with the sham group. ^#^ *p* < 0.05, ^##^ *p* < 0.01, ^###^ *p* < 0.001 compared with the TBI + veh group

Levels of mRNA for the cytokines in sham animals were determined to be low, in accord with the unchallenged status of the brain. However, on evaluation 8 h after TBI, the expression of TNF-α mRNA was significantly elevated compared to sham animals; the fold change was +39 ± 4, *p* < 0.001, Fig. 6a. The levels of expression of IL-1β and IL-6 were also significantly elevated compared to sham levels (+63 ± 8-fold and +1275 ± 261-fold, respectively, *p* < 0.001, Fig. 6 b, c). Pom significantly decreased the expression of all pro-inflammatory cytokine mRNA levels. The percentage reduction from the TBI-veh levels for each cytokine was determined to be, for TNF-α (78 %, *p* < 0.001), for IL-1β (50 %, *p* < 0.05), and for IL-6 (52 %, *p* < 0.01).

We also compared the cortical protein abundances of TNF-α, IL-1β, and IL-6 in sham, vehicle-treated and Pom-treated TBI groups (Fig. 6 d–f). Basal protein levels were low in the cortical tissue of the sham group. Eight hours following TBI, cytokine protein levels in cortex were significantly elevated in the vehicle-treated group (*p* < 0.001): TNF-α 146 ± 15 pg/mg of total protein, IL-1β 356 ± 12 pg/mg, and IL-6 531 ± 46 pg/mg. Pom significantly reduced the TBI-induced increase in cortical levels of all three pro-inflammatory cytokines. Specifically, these protein levels in Pom-treated TBI rats were lowered by 45 % for TNF-α (*p* < 0.001), 31 % for IL-1β (*p* < 0.001), and 30 % for IL-6 (*p* < 0.001), as compared to TBI-veh tissue samples.

### Primary cortical mixed cell cultures studies: Pom protects neurons and attenuates the activation of microglial cells from glutamate-induced excitotoxic 24 h stress

**Fig. 7:**
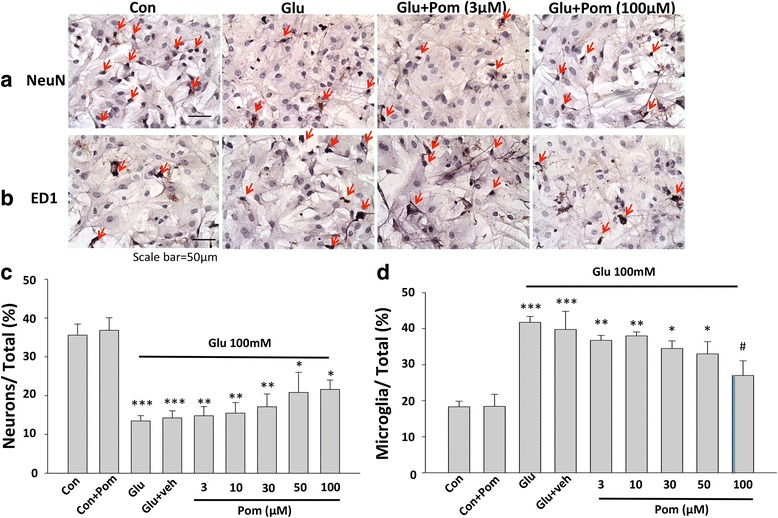
Pom protected neurons and attenuated the activation of microglial cells from glutamate-induced excitotoxicity 24 h after a challenge with glutamate. **a**, **b** Photomicrographs of the immunochemical staining for NeuN-positive (+) neurons and CD68+ microglial cells are provided. **a** Numbers of NeuN+ cells were significantly reduced by treatment with glutamate (100 mM) compared to control (Con) cultures. The addition of Pom (3–100 μM), 30 min after a challenge with glutamate led to a dose-dependent attenuation in NeuN+ cell loss. *Arrows* indicate NeuN+ neurons (*brown color*) in control, glutamate, and glutamate + Pom (3 or 100 μM) treated cultures. **b** Staining for CD68+ microglial cells in control cultures, cultures treated with glutamate (100 mM), cultures treated with glutamate (100 mM), and Pom (3 or 100 μM) are shown. A challenge with glutamate significantly elevated the numbers of CD68+ cells, treatment with Pom (3–100 μM) significantly attenuated the activation of microglial cells in a dose-dependent manner. *Arrows* indicate microglia (*brown color*). **c**, **d** Quantitative summaries of the effects of glutamate and Pom treated cultures on NeuN+ cells and activated microglial cells are provided. Mean ± S.E.M. (*n* = 8 in each group). *p < 0.05, **p < 0.01, ***p < 0.001 compared with control cultures; ^#^ *p* < 0.05 compared with glutamate alone challenged cultures. *Scale bar* = 50 μm

Immunocytochemical studies showed that 24 h following a challenge with glutamate (100 mM), the proportion of neurons (NeuN+ cells) in a cortical mixed cell culture declined from 36 ± 3 % control cultures to 14 ± 1 % in glutamate-alone challenged cultures (*p* < 0.001) (representing a 61 % loss of the total neuron number). However, when Pom was added to the cultures 30 min after glutamate, there was a concentration-dependent attenuation of glutamate-induced neuron loss (Pom 3, 10, 30, 50, and 100 μM; Fig. 7 a, c), with the highest dose rescuing 37 % of dying neurons. Microglial cell activation was elevated by glutamate (100 mM) challenge, as indicated by elevated numbers of CD68+ cells (2.25-fold greater than control levels). Pom attenuated the proportion of activated microglia, reaching statistical significance at a Pom concentration of 100 μM (*p* < 0.05 versus the glutamate alone group; Fig. 7 b, d) (reducing glutamate-induced microglial activation by 64 %).

### Glutamate-induced microglial generation of TNF-α protein measured in culture media over time is mitigated by Pom

**Fig. 8:**
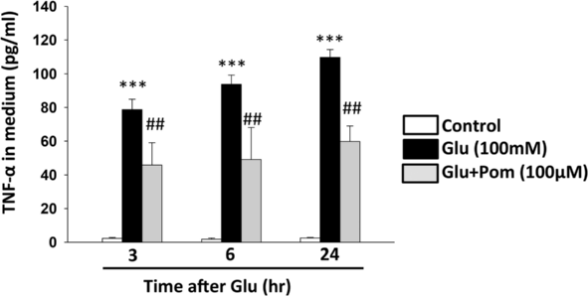
Treatment with Pom significantly attenuated a glutamate-induced excitotoxicity-induced elevation of TNF-α protein in primary mixed cortical culture media. Treatment with Pom (100 μM), 30 min after the addition of glutamate (100 mM for 24 h) significantly reduced the levels of TNF-α protein detected in culture media. Media samples were obtained at 3, 6, and at 24 h after challenge with glutamate. Mean ± S.E.M. (*n* = 8 or 6 in each group). ***p < 0.001 compared with control cultures. ^##^ *p* < 0.01 compared with glutamate alone treated cultures

Based upon the optimal efficacious concentration of Pom observed from the glutamate study, a similar 100 μM Pom concentration was evaluated for actions on TNF-α generation. As illustrated in Fig. 8, glutamate (100 mM) challenge of cortical mixed cell cultures resulted in microglial cell activation and the time-dependent synthesis and release of elevated TNF-α protein levels into the culture media. Specifically, TNF-α levels rose from a resting concentration of 3 to 110 pg/ml at 24 h after glutamate challenge. Pom significantly attenuated the effects of glutamate at all times evaluated during the study, reducing TNF-α levels in media by 45 % (Fig. 8).

## Discussion

TBI is typically subdivided into time-dependent components. This consists of an initial primary injury that in the present study involves a focal, physical deformation of the dura mater, and a series of secondary events that include inflammatory, oxidative stress, and excitotoxicity response-related pathology [39]. In the present study, we used a well-characterized controlled cortical impact model of TBI. In this model, the primary injury, depending on the severity, typically leads to the formation over time of a necrotic core that is not amenable to pharmacological manipulation [39–41]. However, the molecular effectors responsible for the later, secondary components of TBI do hold the potential for beneficial drug intervention. Prior studies of others and ourselves utilizing p53 inactivators as pharmacological tools to halt programmed cell death processes indicate that tissues in the marginal zone of the focal TBI microenvironment may be compromised but not yet destined to become irreversibly dysfunctional or apoptotic [28, 42]. These are the tissues predicted most likely to respond to drug treatment strategies that may enhance cell survival [39]. Key players in secondary damage are cytokine, pro-inflammatory mediators of inflammation such as TNF-α, play a significant role in directing cell death pathways, and, interestingly and somewhat counterintuitively, also have a role in cell survival pathways [43–45]. TBI-induced activation of brain immune cells can induce upregulations of TNF-α protein that can amplify additional pathologies associated with secondary excitotoxicity-related injury such as glutamate release from astrocytes, which can additionally cause blood-brain barrier dysfunction and further drive disease processes [17, 46]. The unchecked generation of TNF-α may induce a self-propagating pathological cascade of neuroinflammation and neuronal loss [47] that upregulates multiple cytokines downstream of the TNF-α receptor, such as IL-6, IL-1β, and TNF-α [48]. Evidence of such elevations are documented in clinical and experimental TBI CSF and serum from individuals with clinical brain injuries and in rodent models with experimental brain injuries [49–51]. Not surprisingly, numerous animal studies have adopted anti-inflammatory approaches with drug benefits having been observed in various models of TBI, giving added weight to the significance of inflammation in the secondary pathology of TBI [20, 51–55]. These observations indicate that inflammation is an interesting target for study and potentially drug candidate development.

Pom is a third generation derivative of thalidomide (*N*-α-phthalimidoglutarimide). Thalidomide, first used in the clinic in the late 1950s as a non-addictive, non-barbiturate sedative and then found effective as an anti-emetic in women suffering from pregnancy-associated nausea, notably was associated with birth defects and was rapidly removed from clinical use in the early 1960s [56–58]. Following the failure of other drugs, the fortuitous use in 1964 of thalidomide as a sedative in a patient suffering leprosy and the remarkable resolution of the associated symptoms [59] provided the initial insight of the compound’s immunomodulatory actions. This spurred the use and eventual approval of thalidomide as a treatment of erythema nodosum leprosum, documenting its now well-characterized anti-inflammatory actions [60], and the development of structure/activity relations for the creation of more potent analogs. Moreira and co-workers determined that thalidomide’s anti-inflammatory properties are driven by the drug’s ability to reduce the half-life of TNF-α mRNA, thereby lowering protein levels of this potent pro-inflammatory cytokine [61, 62]. These initial studies gave rise to the development of multiple thalidomide-based immunomodulatory drugs, two of which (Lenalidomide and Pom) are presently used clinically for multiple myeloma and other cancers [63].

Our previous side-by-side evaluation of thalidomide, lenalidomide, and Pom in an established mouse retinal explant outgrowth assay [64] demonstrated that Pom, unlike its other analogs, was not associated with neurotoxicity at anti-inflammatory concentrations. These studies, together with our evaluation of thalidomide in a mouse transgenic model of Alzheimer’s disease in which it lacked efficacy and failed to lower elevated brain levels of TNF-α at a clinically translatable dose [65], focused our attention to more potent anti-inflammatory analogs [21], including Pom. The findings of our present study indicate that Pom possesses beneficial properties across a broad series of outcome measures in a well-characterized animal model of a penetrating form of TBI. A single administration of Pom (0.5 mg/kg, i.v.) at 5 h but not 7 h after the induction of TBI attenuated the size of the resulting cortical lesion, reduced the levels of apoptotic and degenerating neurons, and, notably, improved multiple motor functional outcomes evaluated 24 h after injury. Additionally, measures of TBI-upregulated cortical mRNA and protein levels of the pro-inflammatory cytokines TNF-α, IL-1β, and IL-6 were significantly blunted by Pom. In vitro studies undertaken in mixed cell primary cortical cultures assessing the effects of Pom on the excitotoxic stressor glutamate, thought to be important in the cascade leading to secondary TBI pathology, illustrated both neuroprotective and anti-inflammatory (TNF-α lowering) activities that may underpin our observed activity of Pom in vivo. These data strongly support the further preclinical characterization of this FDA-approved immunomodulatory anti-cancer drug as a potential treatment for TBI in the clinic.

This study may be the first to demonstrate that Pom possesses efficacious neuroprotective properties that, based upon our data, is mediated via an anti-inflammatory mechanism of action on brain glial cells. This property is in accord with our prior evaluation of 3,6′-dithiothalidomide, a structurally related and likewise more potent TNF-α lowering analog of thalidomide [66]. It has been suggested that the timing of anti-inflammatory therapies with regard to the initial injury may prove to be critical for the determination of beneficial over harmful longer term drug effects [67, 68]; thus, great care must be taken in designing any therapeutic agent treatment regimen. Studies in mice generated to either lack TNF-α or its receptors suggest that elevated TNF-α is damaging during the acute period following a TBI but is involved in regenerative processes during the later chronic post-injury phase [54, 69, 70]. In particular, signaling via TNF-α receptor 1 (p55) has been implicated in the immediate deleterious actions of injury-induced acute TNF-α release, with knockout mice demonstrating a reduced contusion volume and improved neurobehavioral performance for up to 4 weeks following CCI TBI versus wild-type mice [71]. In contrast, TNF-α receptor 2 (p75) knockout mice demonstrated exacerbated post-injury [68], suggesting an involvement of this receptor in the later tissue repair.

The implication from our study is that the window of therapeutic intervention for Pom is up to and including 5 h after TBI and appears to close by 7 h. Notably, this is in accord with that observed in a recent study in which cellular apoptosis was blocked by the use of pharmacological p53 inactivators to define the cellular population that are amenable to rescue [28]. It may be the case that if a larger Pom dose-effect study had been undertaken, a different drug dose-dependent therapeutic window relationship—possibly longer—may have been identified. This possibility requires further characterization to be proven or disproven. Our previous studies inhibiting TNF-α synthesis in a milder form of TBI, involving a weight drop concussive injury in mice, demonstrated a therapeutic window of 12 h that closed within 18 h [20]; this suggests a relationship between TBI severity and the point at which the secondary phase of brain injury can be halted or reversed.

Strengths of the controlled cortical impact TBI model system in rodent are the reproducibility of the injury and the readily measurable changes in tissue morphology subsequent to injury, a measure amenable to the detection of beneficial therapeutic manipulation. Additional features of the model that align with TBI seen in the clinical setting include disruption of the blood-brain barrier and the recruitment of peripheral blood mononuclear cells that, taken together, create a useful model for the study of penetrating injuries of the dura mater and candidate drug treatments. The observed alterations in rodent motor function and cortical cellular and histological measures were not associated with any TBI- or Pom-induced lowering of animal body temperature, a confound in studies of beneficial influences on neuronal survival following a TBI [71, 72]. A potential caveat of the present study is that experimental animals were euthanized 24 h after the induction of the brain injury, which is in line with prior TBI and stroke studies by others and ourselves [28, 73, 74]. Nonetheless, significant attenuations of TBI-induced pathology and functional measures were observed during this relatively short time frame—suggestive of more long-term drug benefits after TBI. However, further studies will need to be performed to confirm this speculation. As important, longer term studies would allow evaluation of potential adverse drug actions (for example, peripheral neuropathy and/or immunosuppression—which were not evaluated after our single dose short-term study).

Pom has been investigated in various rodent models of human disease including peripheral blood cell cancer, brain and colorectal cancer, and models of dermal fibrosis [75–80]. The doses of Pom utilized in such studies ranged from 0.3 up to 50 mg/kg body weight administered on daily or on an alternate daily schedule for 2 to 6 weeks. Drug delivered was by the oral or intraperitoneal routes. To our knowledge, our study is the first to evaluate Pom in a model of TBI. Interestingly, the dose of Pom (0.5 mg/kg) used in our study is similar to that used in other experimental investigations [75–80]. An additional insight from the present study is that a single administration of Pom was able to provide significant benefits to a range of outcome measures after TBI. If this feature were to translate to the treatment of human TBI, it may suggest that there could be a reduced occurrence of the documented, undesirable effects associated with prolonged treatment with Pom [25]. The dose and administration regiment of Pom utilized for the clinical treatment of multiple myeloma is 4 mg once daily taken on days 1–21 of repeated 28-day cycles [25], but doses as high as 10 mg have been administered. The equivalent human dose of Pom that matches to that used in our rat TBI study (0.5 mg/kg), when normalized to body surface area across species in accord with FDA guidelines, is approximately 5 mg for a 60- to 65-kg human [81]. This dose is similar to that currently used in clinical practice today.

Until quite lately, the molecular target of thalidomide and its analogs was not known but was recently identified as the protein cereblon [82]. Cereblon has been described as a direct protein target of Pom, and a physical drug-protein interaction appears responsible for the observed immunomodulatory properties of this class of medicines [83], albeit recent studies have demonstrated that Pom can inhibit cytokines in cereblon deficient mice [84]—suggesting potential involvement of additional pathways in some cells. Cereblon mRNA appears to be widely distributed in the adult rodent brain with almost all neurons expressing it, whereas levels observed in astrocytes and associated cells are reported to be the modest to negligible [85]. Cereblon may play several roles in the physiology of brain tissues: impairing the function of certain neuronal cell ion channels, a calcium ion channel (BK_Ca)_ [86], and a chloride channel (CIC-2) [87, 88]. Cereblon also interacts with proteins that bind with damaged DNA, targeting the complexes for ubiquitination and proteasomal degradation [87]. It has been reported to bind with the α-subunit of AMP-activated protein kinase (AMPK) that interferes with the normal function of AMPK, a critical sensor of cellular energetics. Although little is known in relation to cereblon following brain injury, its depletion in mouse embryonic fibroblasts results in resistance to a broad number of cytotoxic insults, including oxidative stress, and knocking out cereblon in mice results in a reduced infarct volume in the middle cerebral occlusion model of stroke [89].

Hence, in addition to the effects of Pom on TNF-α regulation [66], Pom may provide pleiotrophic effects by binding to and sequestering cereblon. Thus, Pom may inhibit potential cereblon-mediated detrimental physiological actions on stressed neurons leading to neuroprotection after TBI. Clearly, more studies will be required to identify any specific effects of Pom and cereblon on TBI-compromised neuronal tissues and in neuroinflammation. Taken together, the present study identifies an interesting candidate agent for development as a treatment of TBI in the clinic—a possible new medicine for a highly prevalent medical condition that presently has none.

## Conclusions

Post-injury treatment with a single clinically translatable dose of Pom within 5 h significantly mitigated functional impairments in a well-characterized animal model of controlled cortical impact TBI. Pom reduced the injury lesion volume, improved neuronal survival, and ameliorated ensuing neuroinflammation. These findings advocate further appraisal and optimization of Pom as a new treatment strategy for clinical TBI.

## Abbreviations

BSA, bovine serum albumin; EBST, elevated body swing test; FJC, fluoro Jade C; mNSS, modified neurological severity score; PS, phospholipid phosphatidylserine; Pom, pomalidomide; TBI, traumatic brain injury; FDA, US Food and Drug Administration; veh, vehicle
