# Erratum to: Pomalidomide mitigates neuronal loss, neuroinflammation, and behavioral impairments induced by traumatic brain injury in rat

**DOI:** 10.1186/s12974-016-0668-6

**Published:** 2016-09-12

**Authors:** Jing-Ya Wang, Ya-Ni Huang, Chong-Chi Chiu, David Tweedie, Weiming Luo, Chaim G. Pick, Szu-Yi Chou, Yu Luo, Barry J. Hoffer, Nigel H. Greig, Jia-Yi Wang

**Affiliations:** 1 Graduate Institute of Medical Science, College of Medicine, Taipei Medical University, 250 Wu-Hsing St., Taipei, 110 Taiwan; 2 Department of Nursing, Hsin Sheng Junior College of Medical Care and Management, Taoyuan, Taiwan; 3 Department of General Surgery, Chi Mei Medical Center, Tainan and Liouying, Taiwan; 4 Drug Design & Development Section, Translational Gerontology Branch, Intramural Research Program, National Institute on Aging, National Institutes of Health, Baltimore, USA; 5 Department of Anatomy and Anthropology, Sackler School of Medicine and Sagol School of Neuroscience, Tel Aviv University, Tel Aviv, Israel; 6 Graduate Program on Neuroregeneration, College of Medical Science and Technology, Taipei Medical University, Taipei, Taiwan; 7 Department of Neurosurgery, Case Western Reserve University School of Medicine, Cleveland, OH USA; 8 Department of Physiology, College of Medicine, Taipei Medical University, 250 Wu-Hsing St., Taipei, 110 Taiwan

## Erratum

Upon publication of the original article [1], it was noticed that Jing-Ya Wang’s name was incorrectly written as ‘Jin-Ya Wang’. This has now been corrected in this erratum and updated in the original article.
